# Fertility differences between two wild-type Drosophila melanogaster lines correlate with differences in the expression of the Jheh1 gene, which codes for an enzyme degrading juvenile hormone

**DOI:** 10.18699/vjgb-24-22

**Published:** 2024-04

**Authors:** O.V. Andreenkova, N.V. Adonyeva, V.M. Efimov, N.E. Gruntenko

**Affiliations:** Institute of Cytology and Genetics of the Siberian Branch of the Russian Academy of Sciences, Novosibirsk, Russia; Institute of Cytology and Genetics of the Siberian Branch of the Russian Academy of Sciences, Novosibirsk, Russia; Institute of Cytology and Genetics of the Siberian Branch of the Russian Academy of Sciences, Novosibirsk, Russia; Institute of Cytology and Genetics of the Siberian Branch of the Russian Academy of Sciences, Novosibirsk, Russia

**Keywords:** Drosophila melanogaster, Wolbachia, jhamt, Jheh1, gene expression, fertility, juvenile hormone metabolism, Drosophila melanogaster, Wolbachia;, jhamt, Jheh1, экспрессия генов, плодовитость, метаболизм ювенильного гормона

## Abstract

Juvenile hormone plays a “status quo” role in Drosophila melanogaster larvae, preventing the untimely metamorphosis, and performs a gonadotropic function in imagoes, ensuring the ovaries’ preparedness for vitellogenesis. The decreased level of juvenile hormone results in reproductive disorders in D. melanogaster females including a delay in the oviposition onset and a fertility decrease. Another factor that can affect the insect reproduction is an infection with the maternally inherited symbiotic α-proteobacterium Wolbachia. The present study is devoted to the analysis of the expression of two juvenile hormone metabolism genes encoding enzymes of its synthesis and degradation, juvenile hormone acid O-methyltransferase ( jhamt) and juvenile hormone epoxide hydrase (Jheh1), respectively, in four wild-type D. melanogaster lines, two of them being infected with Wolbachia. Lines w153 and Bi90 were both derived from an individual wild-caught females infected with Wolbachia, while lines w153T and Bi90T were derived from them by tetracycline treatment and are free of infection. Line Bi90 is known to be infected with the Wolbachia strain wMel, and line w153, with the Wolbachia strain wMelPlus belonging to the wMelCS genotype. It was found that infection with either Wolbachia strain does not affect the expression of the studied genes. At the same time, it was shown that the w153 and w153T lines differ from the Bi90 and Bi90T lines by an increased level of the Jheh1 gene expression and do not differ in the jhamt gene expression level. Analysis of the fertility of these four lines showed that it does not depend on Wolbachia infection either, but differs between lines with different nuclear genotypes: in w153 and w153T, it is significantly lower than in lines Bi90 and Bi90T. The data obtained allow us to reasonably propose that the inter-line D. melanogaster polymorphism in the metabolism of the juvenile hormone is determined by its degradation (not by its synthesis) and correlates with the fertility level.

## Abstract (ru)

Ювенильный гормон играет у личинок Drosophila melanogaster роль “status quo” гормона, препятствуя преждевременному наступлению метаморфоза, а у имаго выполняет гонадотропную функцию, обеспечивая подготовку яичников к вителлогенезу. При снижении уровня ювенильного гормона у самок D. melanogaster наблюдаются нарушения репродукции, выражающиеся в задержке начала откладки яиц и снижении плодовитости. Еще одним фактором, способным повлиять на репродуктивную функцию насекомых, является инфицирование матерински наследуемой симбиотической α-протеобактерией Wolbachia. Настоящее исследование посвящено анализу экспрессии двух генов метаболизма ювенильного гормона, кодирующих ферменты его синтеза и деградации – кислую О-метилтрансферазу ювенильного гормона (jhamt) и эпоксидгидразу ювенильного гормона (Jheh1) соответственно, у четырех линий D. melanogaster дикого типа, две из которых инфицированы Wolbachia. Линии w153 и Bi90 происходят от отдельных самок, отловленных в дикой природе, и инфицированы Wolbachia, а линии w153T и Bi90T получены на их основе посредством тетрациклиновой обработки и характеризуются отсутствием инфекции. Известно, что линия Bi90 инфицирована штаммом Wolbachia генотипа wMel, а линия w153 – штаммом Wolbachia wMelPlus, относящимся к генотипу wMelCS. Обнаружено, что инфицирование как одним, так и другим штаммом Wolbachia не влияет на экспрессию исследованных генов. В то же время показано, что обе линии с ядерным генотипом w153 отличаются от линий с генотипом Bi90 повышенным уровнем экспрессии гена Jheh1 и не различаются по уровню экспрессии гена jhamt. Уровень плодовитости не зависел от инфицирования Wolbachia, но у линий с ядерным генотипом w153 он оказался существенно ниже, чем у линий с ядерным генотипом Bi90. Полученные данные позволяют сделать обоснованное предположение о том, что межлинейный полиморфизм D. melanogaster по метаболизму ювенильного гормона коррелирует с уровнем плодовитости и определяется деградацией гормона, а не его синтезом

## Introduction

According to the current understanding of the genetic control of Diptera reproduction, a key role is played by 20-hydroxyecdysone (20E), while juvenile hormone (JH) only prepares ovaries for vitellogenesis, unlike in most other insect orders, where JH has the function that in Diptera is performed by 20E (Roy et al., 2018; Wu et al., 2021). The balance between these two hormones determines many events in the life of holometabolic insects from the larval period, where 20E initiates the start of moulting, and the JH level determines whether it will be larval moulting (if it is high), or the onset of metamorphosis (if it is low) (Truman, Riddiford, 2007), the neurohormonal stress response, which involves both hormones, and the regulation of changes in ovaries under heat stress or starvation (Gruntenko et al., 2003a; Terashima et al., 2005; Gruntenko, Rauschenbach, 2008).

Despite the secondary role of JH in oogenesis regulation and reproduction control in Drosophila, there are data indicating that in flies with a decreased level of JH, the reproduction process is disrupted, which is expressed as a delay in oviposition onset and a fertility decrease (Altaratz et al., 1991; Gruntenko et al., 2003b; Yamamoto et al., 2013; Meiselman et al., 2017), and endogenic JH treatment of females speeds up egg maturation (Richard et al., 2001). Thus, we can assume that through controlling vitellogenins uptake by oocytes (Berger, Dubrovsky, 2005), JH takes part in the determination of the fertility level in Drosophila.

The intracellular signaling of JH is well described in the literature (Jindra et al., 2015; Roy et al., 2018), including the JH receptor complex Methoprene-tolerant (Met) – Taiman – Germ cell-expressed (Gce), the heat shock protein HSP83 and nucleoporin Nup358, which interact with Met and ensure JH transfer into the nucleus and the activation of the transcriptional factor Kr-h1 by it. At the same time, the mechanisms of the JH level regulation are still underresearched.

To add to the knowledge regarding this subject, we have estimated the level of fertility and expression of the genes responsible for JH synthesis and degradation, jhamt and Jheh1, in four Drosophila melanogaster lines, two of which were earlier demonstrated to differ in the fertility level (Adonyeva et al., 2021). jhamt codes for juvenile hormone acid O- methyltransferase (JHAMT), transforming JH acid or inactive JH precursors into the active form of the hormone at the final stage of JH biosynthesis in insects (Niwa et al., 2008). Jheh1 codes for one of the forms of JH epoxide hydrolase that inactivates the hormone via hydrolysis of the epoxide functional group producing JH diol (Flatt et al., 2005).

Notably, another factor capable of affecting fly fertility as well as JH metabolism is the infection with the maternallyinherited symbiotic α-proteobacterium Wolbachia pipientis (Werren et al., 2008; Burdina, Gruntenko, 2022). Wolbachium is a widely spread intracellular insect symbiont infecting more than 40 % of the studied species and greatly affecting host physiology (Werren et al., 2008; Burdina, Gruntenko, 2022). As lines w153T and Bi90T, the differences in the fertility of which were shown earlier, were derived from lines w153 and Bi90, which, in turn, were derived from single females caught in nature and were initially infected with Wolbachia, we decided to use for analysis lines w153 and Bi90, carrying the infection, and lines w153T and Bi90T, having undergone antibacterial therapy, to search for possible effects of Wolbachia on the fertility level and the expression of the JH metabolism genes

## Materials and methods

Drosophila lines. In the work, we used four D. melanogaster lines: the w153 and Bi90 lines, derived from single females and carrying Wolbachia strains of the wMelCS and wMel genotypes, respectively (Ilinsky, 2013), and their derivatives, w153T and Bi90T, which underwent antibacterial therapy prior to the start of experiments. The lines were received from the collection of the Institute of Cytology and Genetics SB RAS. Notably, the Wolbachia wMelPlus strain, infecting the w153 line, differs from other published strains of wMelCS by a large chromosomal inversion (Korenskaia et al., 2022).

Flies were kept on a standard medium (agar-agar, 7 g/l; corn flour 50 g/l; dry yeast 18 g/l; sugar 40 g/l) in an incubator (Sanyo, Japan) at a temperature of 25 °C, relative humidity of 50 %, and 12:12 h light cycle. For the experiments, flies were synchronized at eclosion (they were collected 3–4 h afterwards). To analyze fertility and gene expression levels, 10-days-old females were taken

Total RNA isolation and real-time RT-PCR. To assess the number of mRNA of the jhamt and Jheh1 genes, 15 females per biological replicate per line were frozen in liquid nitrogen in 1.5 ml Eppendorf tubes. In total, three biological replicates of all four Drosophila lines were performed. After removing the tubes from liquid nitrogen, 150 μl of TRI reagent No. BCBT8883 (Sigma, USA) was added to each tube and flies were homogenized. To remove large tissue fragments from the homogenate, the tubes were centrifuged for 5 min at 10,000 rpm in an Eppendorf centrifuge at a temperature of 7 °С and then the homogenate was transferred to clean 0.5 μl tubes. 30 μl of cold chloroform was added, and after shaking the tubes were left for 15 min at room temperature. Afterwards, the homogenate was centrifuged for 15 min at 12,000 rpm and a temperature of 7 °С. 75 μl of cold isopropanol was added to the supernatant, and after shaking the tubes were left for 10 min at room temperature. After centrifugation (12,000 rpm; 10 min), pellets were washed with 150 μl of 75° ethanol twice with centrifugation in-between, dried and dissolved in 100 μl of deionized water. RNA concentration was measured with the use of Nanodrop OneC (Thermo Scientific, USA) and adjusted to 200 ng/μl by the addition of deionized water. cDNA synthesis was performed with the use of ABScript III RT Master Mix for qPCR with gDNA Remover No. RK20429 (ABclonal Technology, China) in accordance with the manufacturer’s protocol.

jhamt and Jheh1 expression were analyzed with CFX96 Touch amplificator (Bio-Rad, USA) using real-time RT-PCR with the M-427 set with SYBR-Green I (Syntol, Russia). Data were normalized on Act5C. For every sample, three technical replicates were performed. Primer sequences used in the study are presented in Table 1.

**Table 1.**
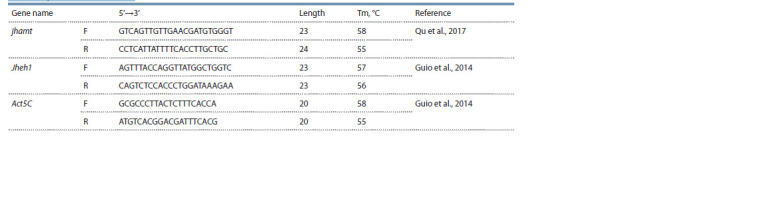
Primer sequences used in RT-PCR

Fertility. To assess fertility, three male-female pairs aged 0–5 h were placed in cultivation vials (10 vials per experimental group), where they were left to lay eggs under standard conditions; flies were transferred to new vials every 24 h for 10 days. Fertility was calculated as the number of offspring (imagoes) eclosed from the eggs laid by experimental flies during the 10th day per one parent female.

Statistical analysis. Statistical significance of the differences in fertility (number of eggs per day per female) in the experimental groups was assessed using Student’s t-test. Pairwise comparisons were performed using the Benjamini– Hochberg correction. In all cases, p <0.05 was considered statistically significant. Histogram data are presented as average means ± SEM

Data on gene expression were analyzed by 2−ΔΔCT method (Livak, Schmittgen, 2001) using three biological replicates, each of which was obtained from three technical ones. Since Real-Time CFX96 Touch amplificator (Bio-Rad) provides only the average mean of three technical replicates and standard mean error, it is impossible to check normality, use non-parametric criteria or bootstrap. However, these data are sufficient to calculate sum of squares of the three replicates.

The general formula for the squared error of the mean:

**Formula. 1.**
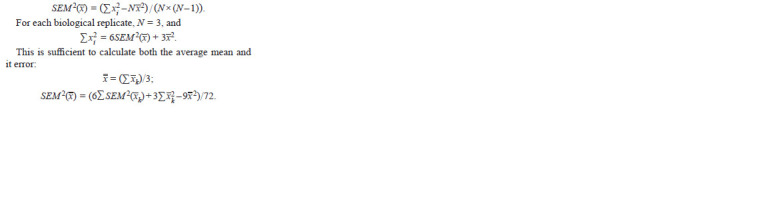
Formula. 1.

The total sum of technical values for each average mean equals 9. When calculating Student’s criterion of the significance of the difference between two average means, 2 × 9 – 2 = 16 degrees of freedom are obtained. It is known that significance criteria like Student’s criterion are resistant to deviations from normality (Kendall, Stewart, 1961) due to the distribution of means approaching normality with increasing sample size.

As we made six comparisons in total, the Benjamini–Hochberg correction was additionally calculated for the p-value to compare with three standard significance levels (Narkevich, Vinogradov, 2020).

## Results and discussion

**Fig. 1.**
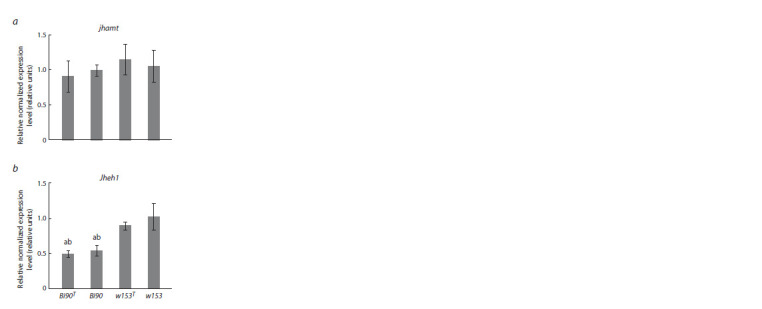
Relative expression level of the jhamt (a) and Jheh1 (b) genes in females of D. melanogaster lines Bi90 (infected with the wMel strain of Wolbachia), w153 (infected with the wMelPlus strain of Wolbachia), Bi90T (uninfected), w153T (uninfected). Each value is the average mean of three biological replicates ± SEM. a – the significance of the differences from females of the w153 line ( p < 0.001); b – the significance of the differences from females of the w153T line ( p < 0.001).

Real-time RT-PCR did not reveal significant differences in the expression level of the gene of juvenile hormone acid O-methyltransferase, jhamt, both between D. melanogaster lines Bi90/Bi90T and w153/w153T (the same genetic background but infected/uninfected with Wolbachia), and between lines Bi90/w153 and Bi90T/w153T (the same infection status but different genetic background) (Fig. 1, a). At the same time, the expression of the JH epoxide hydrolase gene, Jheh1, was significantly decreased ( p < 0.001) in lines Bi90 and Bi90T compared to lines w153 and w153T (see Fig. 1, b). This means that Wolbachia infection has no influence on the expression level of jhamt and Jheh1, which allows us to assume that synthesis and degradation of JH do not change under the bacteria effect. On the other hand, the existing differences in the gene expression level of Jheh1, which encodes an enzyme degrading JH, between D. melanogaster lines Bi90/Bi90T and w153/w153T allow us to assume the existence of differences in the enzyme activity level and, as a result, in JH content, the level of which should be elevated in the Bi90 and Bi90T lines with a decreased expression level of the gene coding for an enzyme degrading the hormone.

**Fig. 2.**
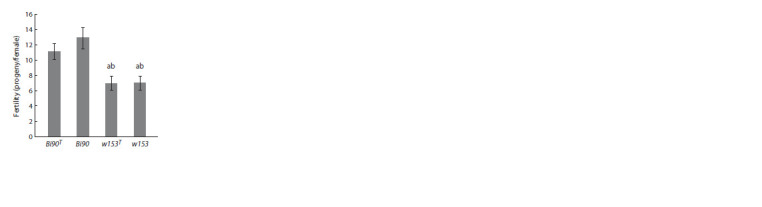
Fertility level of D. melanogaster lines Bi90 (infected with the wMel strain of Wolbachia), w153 (infected with the wMelPlus strain of Wolbachia), Bi90T (uninfected), w153T (uninfected). Each value is the average mean of 10 replicates (three females per replicate) ± SEM. a – the significance of the differences from females of the Bi90 line (p <0.001); b – the significance of the differences from females of the Bi90T line (p < 0.001).

Fertility analysis of the Bi90, Bi90T, w153 and w153T lines revealed that the line with a supposedly lower JH level (w153 and w153T ) are characterized by significantly lower fertility (p < 0.001) compared to both the Bi90 line and the Bi90T line (Fig. 2). This agrees well with earlier data on the correlation of low fertility level with low JH level (Altaratz et al., 1991; Gruntenko et al., 2003b; Yamamoto et al., 2013; Meiselman et al., 2017) or with the Met27 mutation in the gene of the JH receptor (Gruntenko et al., 2000). Data regarding a decrease of the number of germline stem cells in the ovaries of D. melanogaster, carrying mutations in the jhamt and Met genes or with a knockdown of the latter, also indicate the important role JH plays in fertility regulation (Luo et al., 2020).

However, it is worth noting that most researchers attribute the demonstrated disruptions in reproduction and the decrease in fertility to disturbances in JH synthesis or the functioning of its receptor (Altaratz et al., 1991; Yamamoto et al., 2013; Meiselman et al., 2017; Luo et al., 2020), whereas our results indicate a correlation of interline differences in fertility in D. melanogaster with differences in the expression of gene encoding the enzyme that does not synthesize but degrades JH.

The lack of difference in fertility between the lines with the same genetic background infected and the uninfected with Wolbachia (Bi90/Bi90T and w153/w153T ) correlates with the lack of difference in the expression of genes responsible for JH synthesis and degradation and allows us to assume that Wolbachia does not influence this trait. There is a slight contradiction with our earlier data obtained on the Bi90wMelPlus line, where fertility and JH degradation differed from those of the Bi90 line (Gruntenko et al., 2019).

However, it is necessary to note that the Bi90wMelPlus line was received by transferring cytoplasm carrying a Wolbachia strain from the w153 line to the nuclear background of the Bi90 line (via 20 generations of backcrossing females carrying the corresponding Wolbachia strain with males of the Bi90T line), and there is a nonzero probability that some aspects of Wolbachia influence on the physiology of the host can be attributed to the recent bacteria transfer and not merely to its presence in the cytoplasm. This hypothesis is indirectly confirmed by the lack of Wolbachia effect on fertility and JH degradation in the Bi90 line, which was discovered in the same study (Gruntenko et al., 2019).

Additionally, transcriptomic analysis data obtained on infected D. melanogaster females revealed no changes in the differential expression of genes of the JH signaling pathway and the enzymes of its metabolism compared to uninfected females, which correlates with the lack of Wolbachia effect on the expression of the jhamt and Jheh1 genes in D. melanogaster females in our work (Detcharoen et al., 2021; Lindsey et al., 2021).

## Conclusion

To sum up, three reasonable hypotheses could be made based on our data: 1) JH does play a certain part in the regulation D. melanogaster reproduction; 2) JH catabolism has no less, or perhaps more, of a role in providing interline polymorphism by the JH level; 3) Wolbachia does not affect the JH level and fertility in D. melanogaster given the long history of symbiosis between a certain bacterium strain and host line.

## Conflict of interest

The authors declare no conflict of interest.
